# Body Perception and Action Following Deafness

**DOI:** 10.1155/2016/5260671

**Published:** 2016-01-12

**Authors:** M. S. Houde, S. P. Landry, S. Pagé, M. Maheu, F. Champoux

**Affiliations:** École d'Orthophonie et d'Audiologie, Université de Montréal, Montréal, QC, Canada H3N 1X7

## Abstract

The effect of deafness on sensory abilities has been the topic of extensive investigation over the past decades. These investigations have mostly focused on visual capacities. We are only now starting to investigate how the deaf experience their own bodies and body-related abilities. Indeed, a growing corpus of research suggests that auditory input could play an important role in body-related processing. Deafness could therefore disturb such processes. It has also been suggested that many unexplained daily difficulties experienced by the deaf could be related to deficits in this underexplored field. In the present review, we propose an overview of the current state of knowledge on the effects of deafness on body-related processing.

## 1. Introduction

In recent years, increasing attention has been paid to sensory changes in individuals having undergone sensory deprivation. Amongst these investigated populations are the deaf. Deaf individuals can provide a unique insight on the effects of sensory deprivation as some have regained partial hearing through the use of a cochlear implant (CI), a neuroprosthetic device that can restore some level of hearing. The deaf provide opportunities to better understand not only the neuroplasticity underlying sensory deprivation but also the adaptive and maladaptive plasticity that can occur upon recovery of a sensory modality. Current research on the effects of deafness on the perception of the external world suggests that a prolonged period of deafness can lead to significant alterations in sensory processing (for a review, see [1, 2]). Due to the link between perception of the environment and the ability to act in it, several unexplained day-to-day life difficulties observed in the deaf have been proposed as related to deficits in body-related processing (e.g., [3, 4]). Furthering our understanding of the effects of deafness on these processes could thus not only provide insight on the fundamental processes of sensory deprivation but also be of great benefit to individuals living with deafness. The objective of this review is to examine the current state of knowledge on the effects of deafness on body-related processes. In order to provide a well-defined interpretation of the literature, we specifically surveyed nonvisual processing in the deaf, namely, somatosensory, motor, and posture processing. As such, processes that are in direct relation with the visual system (e.g., facial recognition and eye-movement) were not included in the review, even with the existence of a relation with the body.

## 2. Body-Related Processing: Body Image for Perception and Body Schema for Action

The perception-action model proposes that perception of the environment is directly related to the ability to act in it [5]. Influenced by this model, Paillard [6] suggested a distinction between “knowing where” and “knowing how to get there,” implying a difference between the* body image for perception* and the* body schema for action*. A body image consists of the perceptions of one's body (i.e., judgment of bodily properties), while body schema consists of the sensory-motor capacities in which information necessary for movements is integrated, such as for body posture. Thus, in the same way perception differs from movement, body image differs from body schema.

Our body's experience of perception or action is not exclusively limited to the somatosensory system but is accompanied by a variety of body-related inputs. Indeed, there exist no single set of peripheral receptors that inform the brain on the location or self-identity of body parts. The experience of our own body has therefore been shown to be constructed within the central nervous system by the integration of several information sources including somatosensory signals (e.g., [7–10]), visual inputs (e.g. [11–14]), auditory signals (e.g., [15]), and vestibular input (e.g., [16]).

Over the years, several well-known tasks have been developed to directly assess the multiple features related to body image and schema. The investigation of these different body-related processes also include task-dependent effects of bodily illusions. It is understood that the brain's resolution of sensory conflict as induced by bodily illusions is a measurement of the plasticity and flexibility of the underlying body-related processing [17].

Numerous data suggest a significant role for the auditory system in body-related processing. In normally developing individuals, auditory inputs have been shown to interact with the tactile and motor system during speech processing [18, 19], motor behaviour (e.g., [20–24]), posture and balance (e.g., [25–27]), and the general initiation of motor action [28–31]. Finally, the influence of auditory inputs on tactile body perception has also often been demonstrated using multisensory tasks (e.g., [32, 33]). In these audiotactile interaction tasks, the manipulation of auditory input alters tactile perception of either palmar dryness or the number of perceived tactile stimulation.

Evidence suggesting a role of auditory inputs on body-related processing raises important questions for the impact of sensory deprivation [3, 4, 34]. Indeed, considering these evidences, it is perfectly reasonable to expect that deaf individuals would perceive their own body differently than hearing individuals. If so, according to the perception-action model, the deaf would also have altered fundamental perception of their environment.

## 3. Body Image for Perception in the Deaf

### 3.1. Body Sensations

Sight, hearing, taste, smell, and touch are the five traditionally recognized senses. Unlike sight, hearing, smell, and taste, which are all located in specific parts of the body, the sense of touch is much less centralized. Indeed, touch (or the peripheral somatosensory system) is very hard to localize because tactile sensory information enters the nervous system from every area of the body. The sense of touch can provide sufficient information for an individual to determine the numerous features related to a specific object. In this sense, touch allows an individual to learn about the proximal environment and adapt behaviour accordingly. Numerous standardized tasks have been developed to examine human tactile perception. These tasks allow for the examination of detection, resolution, and discrimination capabilities (e.g., static two-point discrimination [35], tactile sensitivity thresholds using Semmes-Weinstein monofilaments [36], and tactile resolution using a grating orientation task [37]).

Similar to studies revealing highly specific changes to visual processing (for a review, see [1, 2, 62, 63]), researches on the tactile domain are often inconsistent depending on the specificity of the tasks used and/or the characteristics of participants (e.g., congenitally deaf; hearing impaired; CI users), suggesting that deafness does not seem to lead to uniform alterations to tactile perception.

Tactile detection and discrimination tasks have been examined in the deaf without statistically significant differences with normally hearing individuals (e.g., [43, 44]). However, a positive correlation between hearing and tactile acuity has been suggested [64]. Additionally, no significant differences were found for tactile detection and discrimination abilities in deaf CI users [45, 46]. More targeted tactile abilities were also investigated and no significant differences were found between early deaf and control groups for spatial sensitivity [48], temporal onset-offset-order discrimination [44], frequency discrimination [49], object identification [38], or tactile discrimination of a rhythmic pattern [42]. However, there is compelling evidence that deafness can result in changes for tactile perception in some specific conditions. For instance, data suggests superior vibrotactile frequency change sensitivity [49] and haptic orientation [47] in congenitally deaf humans. Congenitally deaf CI users were found to have faster reaction time in response to tactile stimuli [40]. However, this increased tactile reaction time was not found in congenitally deaf individuals [39] or in late deaf CI users [40, 41]. The altered tactile abilities from deafness are not exclusively improvements as reduced tactile temporal sensitivity has been revealed in congenitally deaf individuals [48]. These results suggest that, for tactile abilities, plasticity following deafness does not lead to uniform behavioural improvements and can lead certainly to maladaptive behavioural compensation in specific behavioural conditions.

### 3.2. Multisensory Interactions Involving Touch

The sense of touch can be altered through the simultaneous stimulation of another sense. Interaction between senses can enhance overall perceptual accuracy and saliency through cooperative advantages in certain congruent situations (e.g., [65, 66]). Body-related multisensory interactions can be examined through multiple tasks, when the information coming from two modalities are congruent or incongruent. The presentation of conflicting multisensory information can result in an illusory percept. We can gain insight into the ability to integrate multisensory information following deafness by studying alterations to this illusory percept.

#### 3.2.1. Integration of Congruent Auditory and Tactile Information

The interaction between auditory and tactile congruent information has recently been examined in the deaf with CI. Nava et al. [40] showed that both congenitally and late deaf CI users were able to integrate congruent audiotactile stimuli in a reaction time task as effectively as control group members. These results suggest that congenital and acquired deafness does not prevent the development and recovery of this form of basic multisensory processing. However, the authors also found that congenitally deaf CI users (not late deaf CI users) benefited from redundancy gains in the presence of the multisensory stimulation significantly less than their matched controls. This may be explained by a change in tactile perception in those individuals as reviewed in the previous section.

#### 3.2.2. Segregation and Integration of Incongruent Auditory and Tactile Information

Two of the most robust examples of auditory-somatosensory illusions are the “audiotactile illusory flash effect” [33] for the temporal domain and the “parchment skin illusion” [32] for the spectral domain. Both of these tasks are examples of cross-modal interactions. The “audiotactile illusory flash effect” is a nonspeech illusory percept in which the simultaneous presentation of a single somatosensory stimulus with two consecutive sounds can lead to the perception of two distinct tactile sensations in normally hearing individuals. The “parchment skin illusion” is also a nonspeech illusory percept in which an amplification or reduction of high-frequency content from the sound generated by rubbing hands together results in an alteration of the perceived palmar dryness/moistness. Our research team recently use these two tasks to investigated whether a period of deafness disturbed the segregation or the integration of incongruent temporal and spectral audiotactile processing in deaf adults using CI [4, 46]. In both tasks, normally hearing individuals integrated auditory and tactile information effectively in the context of an illusory audiotactile percept, whereas CI users did not. Considering the fundamental nature of the stimuli involved in these tasks, failure to segregate or integrate multisensory information could not be explained by the use of the CI.

## 4. Body Schema for Action in the Deaf

### 4.1. Body Movements

Savelsbergh et al. [51] suggested that the absence of early auditory input could contribute to motor delays in deaf children. This hypothesis was later tested and results suggested that indeed hearing children performed significantly better than deaf children in various evaluations of motor development [50]. More specifically, several studies of motor capacities in deaf children have reported deficits in general dynamic coordination, balance, ball catching abilities, and slower reaction times and speed of movement execution [34, 52, 67, 68]. Interestingly, studies of motor coordination combining deaf and CI users do not report significant differences between deaf and hearing abilities [50].

Several findings suggest that profound deafness may result in disturbances to nonauditory abilities related to serial-order information [54, 56]. In particular, Conway et al. [54] reported deficits of implicit learning abilities in deaf children with CI on color-sequences task. These authors proposed that exposure to sound, a temporally arrayed signal, provides important experience with learning sequential patterns in the environment. This lack of experience with sound at a young age may therefore delay the development of domain-general processing skills of sequential patterns, including nonauditory abilities [55]. In terms of motor sequencing specifically, Schlumberger et al. [53] found that deaf children showed delays in the development in the production of sequential limb movement. Another recent investigation with deaf children with CI by Conway et al. [54] revealed disturbances in the ability to perform a simple fingertip-tapping task. Our research team recently investigate the procedural learning skills of deaf adults with and without CI [56]. The serial reaction time task (SRTT [69]), a task sensitive to both explicit and implicit learning, was administered to investigate possible motor alteration subsequent to auditory deprivation. Results revealed statistically significant differences between the deaf and control groups in sequence-specific learning, with deaf subjects being less efficient than controls in acquiring sequence-specific knowledge. These results further supported impaired sequential learning abilities in the deaf [54, 55].

### 4.2. Body Posture

Researchers have known for more than a century that changes in limb posture (such as crossing the hands) can impair people's performance in temporal order judgments tasks involving tactile stimulus presented to either hand (e.g., [70]). This crossed hands deficit has been attributed to a conflict between externally (i.e., visual and auditory) and anatomically anchored reference systems (i.e., somatosensory) when people localize tactile stimuli [71–73]. Considering this, it has been suggested that such modulation in the perception of touch caused by body posture could be impaired in individuals deprived of one external sensory system, such as in deaf or blind individuals [71]. Indeed, the performance of congenitally blind adults does not seem to be affected by crossing the hands unlike in seeing individuals [71]. This provides insight on the critical role of visual inputs in modulating the perception of touch that may arise from the emergence of specific crossmodal links during development. However, the role of auditory inputs in the development and maintenance of this crucial processing is still unexplored.

Body posture has, however, been evaluated during balance task with a force platform in participants with sensorineural hearing loss. Results suggest that participants with sensorineural hearing loss have poorer balance than normal hearing participants [57–60] and tend to depend mostly on vision and somatosensory inputs [57, 59] to maintain their balance. No significant change in body posture has been revealed for deaf participants with unilateral or bilateral CI [61].

## 5. Discussion

The objective of this review was to survey the existing corpus of research on the effect of deafness on body-related abilities. We also considered studies of body-related abilities in CI users since sensory deprivation, even temporary, can have an effect on the remaining senses. Multiple investigations have examined the effects of sensory deprivation on the remaining senses. Indeed, the effects of deafness on visual abilities have received considerable attention [62, 63], but body-related abilities have garnered considerably less. However, the effect of deafness on body-related processing has important repercussions as it is suggested to be a contributing factor in the daily difficulties observed in the deaf (e.g., [3, 4]).

Auditory inputs are believed to play an important role in the development of body-related processing in the hearing (e.g., [15, 18–33]) and it has been suggested that deafness could have a dramatic impact on these processes [3, 4, 34].

There does not appear to be a global trend on the effects of deafness body-related processes (for an overview of the reviewed articles, see Table 1). The variability between studies surveyed in this review highlights the existing debate over the identity of the altered systems and the mechanisms that mediate adaptive or maladaptive neuroplastic changes following deafness. As shown by Table 1, comparing results between studies is made particularly difficult in the deaf due to the multiple confounding factors involved in deafness. Beyond the categorization of early and late deaf and cochlear implantation, factors such as duration of deafness [74], communication strategy [75], onset of deafness [74, 76, 77], hearing aid use [78], and duration of CI use [46, 79–82] can all influence performance in the deaf. Comparing investigations across studies is complicated by this large set of variables that are often left unreported. Yet, the factors that may constrain or promote performance in body-related processing following deafness are still unknown.

Future research looking at deafness and body-related processes could help further identify the role of auditory experience, whether in early- or late-life, in modulating such processes. These investigations will help deepen our knowledge of not only the neuroplastic changes of deafness to body-processes but also the effects of auditory restoration. More specifically, such understanding will help to identify the systems that are altered and the mechanisms and factors that mediate adaptive or maladaptive changes following deafness. The results from these investigations will provide complementary information to the existing research examining the role of auditory input on external processing following deafness (for a review, see [1, 2]). Moreover, further investigations in this burgeoning field of research will provide additional understanding to the daily difficulties observed in the deaf. Much of the understanding of our surrounding occurs in a multisensory environment in which sensory-motor and auditory cues are present. Identifying behavioural changes in deaf and CI users has direct and significant implications for recognizing the difficulties experienced in day-to-day life. Knowledge stemming from such research will allow more effective patient counselling and expectation management and enable more individualized postimplantation rehabilitation strategies.

## Figures and Tables

**Table 1:** Body-related abilities for deaf and hearing individuals.

Task	Findings^1,2^	References
*Body sensations*		
Object identification	ED = H	[38]
		
Reaction time	ED = H	[39]
	EDCI > H	[40]
	LDCI = H	[40, 41]
		
Discrimination of rhythmic pattern	ED = H	[42]
		
Sensitivity	D = H	[43, 44]
	CI = H	[4, 45, 46]
		
Orientation detection	ED > H	[47]
	CI = H	[4, 46]
		
Temporal sensitivity	ED < H	[48]
Spatial sensitivity	ED = H	[48]
Temporal onset- offset-order discrimination	ED = H	[44]
		
Frequency discrimination	ED = H	[49]
	CI = H	[4, 46]
		
Frequency change detection	ED > H	[49]

*Multisensory interactions involving touch*		
Audiotactile reaction time	CI = H	[40]
Audiotactile segregation	CI ≠ H	[4]
Audiotactile integration	CI ≠ H	[46]

*Body movement*		
Motor coordination	D^*∗*^ = H	[50]
	D < H	[34, 51, 52]
		
Sequential limb movement	D^*∗*^ < H	[53]
		
Serial-order learning	CI < H	[54, 55]
	D^*∗*^ < H	[56]

*Body posture*		
Posture	D^*∗*^ < H	[57–61]

^1^D: deaf, ED: early deaf, LD: late deaf, CI: cochlear implant users, EDCI: early deaf cochlear implant users, LDCI: late deaf cochlear implant users, and D^*∗*^: deaf and cochlear implant users confounded.

^2^D = H, no population difference; D > H, deaf group demonstrating enhanced body related abilities compared to hearing group; D < H, deaf group displaying worse body related abilities compared to hearing group; D ≠ H, deaf group displaying significantly altered abilities compared to hearing group.

## References

[1] Bavelier D., Neville H. J. (2002). Cross-modal plasticity: where and how?. *Nature Reviews Neuroscience*.

[2] Collignon O., Champoux F., Voss P., Lepore F. (2011). Sensory rehabilitation in the plastic brain. *Progress in Brain Research*.

[3] Nasir S. M., Ostry D. J. (2008). Speech motor learning in profoundly deaf adults. *Nature Neuroscience*.

[4] Landry S. P., Guillemot J.-P., Champoux F. (2013). Temporary deafness can impair multisensory integration: a study of cochlear-implant users. *Psychological Science*.

[5] Milner A. D., Goodale M. A. (1995). *The Visual Brain in Action*.

[6] Paillard J., Gantchev G. N., Mori S., Massion J. (1999). Body schema and body image: a double dissociation in deafferented patients. *Motor Control: Today and Tomorrow*.

[7] Lackner J. R. (1988). Some proprioceptive influences on the perceptual representation of body shape and orientation. *Brain*.

[8] Ramachandran V. S., Hirstein W. (1998). The perception of phantom limbs. The D. O. Hebb lecture. *Brain*.

[9] Naito E., Roland P. E., Ehrsson H. H. (2002). I feel my hand moving: a new role of the primary motor cortex in somatic perception of limb movement. *Neuron*.

[10] Ehrsson H. H., Kito T., Sadato N., Passingham R. E., Naito E. (2005). Neural substrate of body size: illusory feeling of shrinking of the waist. *PLoS Biology*.

[11] Petkova V. I., Ehrsson H. H. (2008). If I were you: perceptual illusion of body swapping. *PLoS ONE*.

[12] Ehrsson H. H. (2007). The experimental induction of out-of-body experiences. *Science*.

[13] Ehrsson H. H., Spence C., Passingham R. E. (2004). That's my hand! Activity in premotor cortex reflects feeling of ownership of a limb. *Science*.

[14] Graziano M. S. A., Cooke D. F., Taylor C. S. R. (2000). Coding the location of the arm by sight. *Science*.

[15] Graziano M. S. A., Reiss L. A. J., Gross C. G. (1999). A neuronal representation of the location of nearby sounds. *Nature*.

[16] Pfeiffer C., Serino A., Blanke O. (2014). The vestibular system: a spatial reference for bodily self-consciousness. *Frontiers in Integrative Neuroscience*.

[17] Kammers M. P. M., van der Ham I. J. M., Dijkerman H. C. (2006). Dissociating body representations in healthy individuals: differential effects of a kinaesthetic illusion on perception and action. *Neuropsychologia*.

[18] Nasir S. M., Ostry D. J. (2009). Auditory plasticity and speech motor learning. *Proceedings of the National Academy of Sciences of the United States of America*.

[19] Ito T., Ostry D. J. (2012). Speech sounds alter facial skin sensation. *Journal of Neurophysiology*.

[20] Gherri E., Driver J., Eimer M. (2008). Eye movement preparation causes spatially-specific modulation of auditory processing: new evidence from event-related brain potentials. *Brain Research*.

[21] Garg A., Schwartz D., Stevens A. A. (2007). Orienting auditory spatial attention engages frontal eye fields and medial occipital cortex in congenitally blind humans. *Neuropsychologia*.

[22] Rorden C., Driver J. (1999). Does auditory attention shift in the direction of an upcoming saccade?. *Neuropsychologia*.

[23] Schaefer K.-P., Süss K.-J., Fiebig E. (1981). Acoustic-induced eye movements. *Annals of the New York Academy of Sciences*.

[24] Van Grootel T. J., Van Opstal A. J. (2009). Human sound-localization behaviour after multiple changes in eye position. *European Journal of Neuroscience*.

[25] Kapoula Z., Yang Q., Lê T.-T. (2011). Medio-lateral postural instability in subjects with tinnitus. *Frontiers in Neurology*.

[26] Park S. H., Lee K., Lockhart T., Kim S. J. (2011). Effects of sound on postural stability during quiet standing. *Journal of NeuroEngineering and Rehabilitation*.

[27] Kanegaonkar R. G., Amin K., Clarke M. (2012). The contribution of hearing to normal balance. *Journal of Laryngology and Otology*.

[28] Valls-Solé J., Solé A., Valldeoriola F., Muñoz E., Gonzalez L. E., Tolosa E. S. (1995). Reaction time and acoustic startle in normal human subjects. *Neuroscience Letters*.

[29] Oude Nijhuis L. B., Janssen L., Bloem B. R. (2007). Choice reaction times for human head rotations are shortened by startling acoustic stimuli, irrespective of stimulus direction. *Journal of Physiology*.

[30] Queralt A., Weerdesteyn V., van Duijnhoven H. J. R., Castellote J. M., Valls-solé J., Duysens J. (2008). The effects of an auditory startle on obstacle avoidance during walking. *Journal of Physiology*.

[31] Reynolds R. F., Day B. L. (2007). Fast visuomotor processing made faster by sound. *The Journal of Physiology*.

[32] Jousmäki V., Hari R. (1998). Parchment-skin illusion: sound-biased touch. *Current Biology*.

[33] Hötting K., Röder B. (2004). Hearing cheats touch, but less in congenitally blind than in sighted individuals. *Psychological Science*.

[34] Wiegersma P. H., Van der Velde A. (1983). Motor development of deaf children. *Journal of Child Psychology and Psychiatry*.

[35] Warwick D., Dunn R., Melikyan E., Vadher J. (2009). *Hand Surgery*.

[36] Bell-Krotoski J. A., Tomancik E. (1987). Repeatability of testing with the Semmes-Weinstein monofilaments. *Journal of Hand Surgery*.

[37] Van Boven R. W., Johnson K. O. (1994). The limit of tactile spatial resolution in humans: grating orientation discrimination at the lip, tongue, and finger. *Neurology*.

[38] Schiff W., Dytell R. S. (1971). Tactile identification of letters: a comparison of deaf and hearing childrens' performances. *Journal of Experimental Child Psychology*.

[39] Heimler B., Pavani F. (2014). Response speed advantage for vision does not extend to touch in early deaf adults. *Experimental Brain Research*.

[40] Nava E., Bottari D., Villwock A. (2014). Audio-tactile integration in congenitally and late deaf cochlear implant users. *PLoS ONE*.

[41] Hauthal N., Debener S., Rach S., Sandmann P., Thorne J. D. (2015). Visuo-tactile interactions in the congenitally deaf: a behavioral and event-related potential study. *Frontiers in Integrative Neuroscience*.

[42] Rosenstein J. (1956). Tactile perception of rhythmic patterns in normal, blind, deaf and aphasic children. Independent studies and capstones. *Paper*.

[43] Donahue A. M., Letowski T. (1985). Vibrotactile performance by normal and hearing-impaired subjects using two commercially available vibrators. *International Journal of Audiology*.

[44] Moallem T. M., Reed C. M., Braida L. D. (2010). Measures of tactual detection and temporal order resolution in congenitally deaf and normal-hearing adults. *Journal of the Acoustical Society of America*.

[45] Conway C. M., Karpicke J., Anaya E. M., Henning S. C., Kronenberger W. G., Pisoni D. B. (2011). Nonverbal cognition in deaf children following cochlear implantation: motor sequencing disturbances mediate language delays. *Developmental Neuropsychology*.

[46] Landry S. P., Guillemot J.-P., Champoux F. (2014). Audiotactile interaction can change over time in cochlear implant users. *Frontiers in Human Neuroscience*.

[47] Van Dijk R., Kappers A. M. L., Postma A. (2013). Superior spatial touch: improved haptic orientation processing in deaf individuals. *Experimental Brain Research*.

[48] Bolognini N., Cecchetto C., Geraci C., Maravita A., Pascual-Leone A., Papagno C. (2012). Hearing shapes our perception of time: temporal discrimination of tactile stimuli in deaf people. *Journal of Cognitive Neuroscience*.

[49] Levänen S., Hamdorf D. (2001). Feeling vibrations: enhanced tactile sensitivity in congenitally deaf humans. *Neuroscience Letters*.

[50] Gheysen F., Loots G., Van waelvelde H. (2008). Motor development of deaf children with and without cochlear implants. *Journal of Deaf Studies and Deaf Education*.

[51] Savelsbergh G. J. P., Netelenbos J. B., Whiting H. T. A. (1991). Auditory perception and the control of spatially coordinated action of deaf and hearing children. *Journal of Child Psychology and Psychiatry and Allied Disciplines*.

[52] Hartman E., Houwen U., Visscher C. (2011). Motor skill performance and sports participation in deaf elementary school children. *Adapted Physical Activity Quarterly*.

[53] Schlumberger E., Narbona J., Manrique M. (2004). Non-verbal development of children with deafness with and without cochlear implants. *Developmental Medicine and Child Neurology*.

[54] Conway C. M., Pisoni D. B., Anaya E. M., Karpicke J., Henning S. C. (2011). Implicit sequence learning in deaf children with cochlear implants. *Developmental Science*.

[55] Conway C. M., Pisoni D. B., Kronenberger W. G. (2009). The importance of sound for cognitive sequencing abilities: the auditory scaffolding hypothesis. *Current Directions in Psychological Science*.

[56] Lévesque J., Théoret H., Champoux F. (2014). Reduced procedural motor learning in deaf individuals. *Frontiers in Human Neuroscience*.

[57] Suarez H., Angeli S., Suarez A., Rosales B., Carrera X., Alonso R. (2007). Balance sensory organisation in children with profound hearing loss and cochlear implants. *International Journal of Pediatric Otorhinolaryngology*.

[58] Cushing S. L., Chia R., James A. L., Papsin B. C., Gordon K. A. (2008). A test of static and dynamic balance function in children with cochlear implants: the vestibular olympics. *Archives of Otolaryngology—Head & Neck Surgery*.

[59] Huang M.-W., Hsu C.-J., Kuan C.-C., Chang W.-H. (2011). Static balance function in children with cochlear implants. *International Journal of Pediatric Otorhinolaryngology*.

[60] de Sousa A. M. M., de França Barros J., de Sousa Neto B. M. (2012). Postural control in children with typical development and children with profound hearing loss. *International Journal of General Medicine*.

[61] Eustaquio M. E., Berryhill W., Wolfe J. A., Saunders J. E. (2011). Balance in children with bilateral cochlear implants. *Otology and Neurotology*.

[62] Bavelier D., Dye M. W. G., Hauser P. C. (2006). Do deaf individuals see better?. *Trends in Cognitive Sciences*.

[63] Pavani F., Bottari D., Murray M. M., Wallace M. T. (2012). Visual abilities in individuals with profound deafness a critical review. *The Neural Bases of Multisensory Processes*.

[64] Frenzel H., Bohlender J., Pinsker K. (2012). A genetic basis for mechanosensory traits in humans. *PLoS Biology*.

[65] Calvert G. A., Thesen T. (2004). Multisensory integration: methodological approaches and emerging principles in the human brain. *Journal of Physiology Paris*.

[66] Stein B. E., Stanford T. R. (2008). Multisensory integration: current issues from the perspective of the single neuron. *Nature Reviews Neuroscience*.

[67] Gayle G. W., Pohlman R. L. (1990). Comparative study of the dynamic, static, and rotary balance of deaf and hearing children. *Perceptual and Motor Skills*.

[68] Siegel J. C., Marchetti M., Tecklin J. S. (1991). Age-related balance changes in hearing-impaired children. *Physical Therapy*.

[69] Perez M. A., Wise S. P., Willingham D. T., Cohen L. G. (2007). Neurophysiological mechanisms involved in transfer of procedural knowledge. *Journal of Neuroscience*.

[70] Burnett C. T. (1904). Studies on the influence of abnormal position upon the motor impulse. *Psychological Review*.

[71] Röder B., Rösler F., Spence C. (2004). Early vision impairs tactile perception in the blind. *Current Biology*.

[72] Röder B., Rösler F., Calvert G., Spence C., Stein B. E. (2004). Compensatory plasticity as a consequence of sensory loss. *Handbook of Multisensory Processing*.

[73] Röder B., Föcker J., Hötting K., Spence C. (2008). Spatial coordinate systems for tactile spatial attention depend on developmental vision: evidence from event-related potentials in sighted and congenitally blind adult humans. *European Journal of Neuroscience*.

[74] Lee D. S., Lee J. S., Oh S. H. (2001). Cross-modal plasticity and cochlear implants. *Nature*.

[75] Hirano S., Naito Y., Kojima H. (2000). Functional differentiation of the auditory association area in prelingually deaf subjects. *Auris Nasus Larynx*.

[76] Naito Y., Hirano S., Honjo I. (1997). Sound-induced activation of auditory cortices in cochlear implant users with post- and prelingual deafness demonstrated by positron emission tomography. *Acta Oto-Laryngologica*.

[77] Giraud A. L., Price C. J., Graham J. M., Frackowiak R. S. J. (2001). Functional plasticity of language-related brain areas after cochlear implantation. *Brain*.

[78] Shiell M. M., Champoux F., Zatorre R. J. (2014). Reorganization of auditory cortex in early-deaf people: functional connectivity and relationship to hearing aid use. *Journal of Cognitive Neuroscience*.

[79] Nicholas J. G., Geers A. E. (2006). Effects of early auditory experience on the spoken language of deaf children at 3 years of age. *Ear and Hearing*.

[80] Pantev C., Dinnesen A., Ross B., Wollbrink A., Knief A. (2006). Dynamics of auditory plasticity after cochlear implantation: a longitudinal study. *Cerebral Cortex*.

[81] Damen G. W. J. A., Beynon A. J., Krabbe P. F. M., Mulder J. J. S., Mylanus E. A. M. (2007). Cochlear implantation and quality of life in postlingually deaf adults: long-term follow-up. *Otolaryngology—Head and Neck Surgery*.

[82] Allen M. C., Nikolopoulos T. P., O'Donoghue G. M. (1998). Speech intelligibility in children after cochlear implantation. *American Journal of Otology*.

